# SpemNet: A Cotton Disease and Pest Identification Method Based on Efficient Multi-Scale Attention and Stacking Patch Embedding

**DOI:** 10.3390/insects15090667

**Published:** 2024-09-02

**Authors:** Keyuan Qiu, Yingjie Zhang, Zekai Ren, Meng Li, Qian Wang, Yiqiang Feng, Feng Chen

**Affiliations:** 1 College of Information Science and Technology, Shihezi University, Shihezi 832003, China; nightwish2024@163.com (K.Q.);; 2 Department of Computer Science, University of York, Heslington YO10 5DD, UK; mtv519@york.ac.uk; 3 Department of Computer Science, Durham University, Durham DH1 3LE, UK; qian.wang173@hotmail.com; 4 School of Information, Central University of Finance and Economics, Beijing 100081, China; yiqiang.feng@mail.utoronto.ca; 5 Institute of Health Policy, Management and Evaluation, University of Toronto, Toronto, ON M5S 2E8, Canada

**Keywords:** cotton pest recognition, image classification, attention mechanism, transformer, efficient multi-scale attention, feature fusion, deep learning

## Abstract

**Simple Summary:**

Cotton is a crucial economic crop, but it is often threatened by various pests and diseases during its growth, significantly impacting its yield and quality. Earlier image classification methods often suffer from low accuracy and struggle to perform effectively in complex real-world environments. This paper proposes a novel image classification network named SpemNet, specifically designed for cotton pest and disease recognition. By introducing the Efficient Multi-Scale Attention (EMA) module and the Stacking Patch Embedding (SPE) module, the network enhances the ability to learn local features and integrate multi-scale information, thereby significantly improving the accuracy and efficiency of cotton pest and disease recognition. Extensive experiments conducted on the publicly available CottonInsect and IP102 datasets, as well as a self-collected cotton leaf disease dataset, demonstrate that SpemNet exhibits significant advantages in key metrics such as precision, recall, and F1 score, confirming its effectiveness and superiority in the task of cotton pest and disease recognition.

**Abstract:**

We propose a cotton pest and disease recognition method, SpemNet, based on efficient multi-scale attention and stacking patch embedding. By introducing the SPE module and the EMA module, we successfully solve the problems of local feature learning difficulty and insufficient multi-scale feature integration in the traditional Vision Transformer model, which significantly improve the performance and efficiency of the model. In our experiments, we comprehensively validate the SpemNet model on the CottonInsect dataset, and the results show that SpemNet performs well in the cotton pest recognition task, with significant effectiveness and superiority. The SpemNet model excels in key metrics such as precision and F1 score, demonstrating significant potential and superiority in the cotton pest and disease recognition task. This study provides an efficient and reliable solution in the field of cotton pest and disease identification, which is of great theoretical and applied significance.

## 1. Introduction

Cotton, as one of the world’s major crops, is often attacked by a variety of pests and diseases during its growth cycle, which seriously affects yield [1]. Xinjiang, with its unique geographical location and climatic conditions, produces high-quality cotton. Pests and diseases are among the key factors restricting the yield and quality of cotton, and the limitations of traditional control methods require effective scientific detection and management methods [2,3]. Utilizing image recognition and artificial intelligence technology combined with deep learning methods to accurately detect cotton pests and beneficial insects [4,5,6,7] can improve yield and quality and promote regional economic development.

In recent years, with the continuous development and application of deep learning technology, models based on the transformer architecture have made remarkable achievements in many fields [8,9,10,11]. Among them, Vision Transformer (ViT) [12], as an innovative model that introduces the self-attention mechanism into image processing, has demonstrated powerful performance in tasks such as image classification, target detection, and semantic segmentation. However, the traditional ViT model still faces certain challenges in dealing with local feature learning and multi-scale information integration.

In order to solve these problems, this study proposes a cotton pest and disease recognition method based on efficient multi-scale attention [13] and stacking patch embedding named SpemNet. This method effectively improves the model’s ability to learn local features and multi-scale information, achieving excellent performance in the cotton pest and disease recognition task.

In this paper, we first introduce the structure and key modules of the SpemNet model, then evaluate it performance and conduct comparative experiments on cotton pest and disease identification tasks. In addition, we conduct ablation experiments to verify the effectiveness of the SPE module and the EMA module in improving model performance. Finally, we discuss the limitations of the model and future directions for improvement.

The main contributions of this paper are summarized as follows:

1. By introducing the SPE module, we address the problems of inter-block information loss and difficulty in learning local features in the traditional Vision Transformer model and adopt the method of adding biases of different directions to the image and stacking them before patch embedding, which effectively enhances the model’s ability to learn local features, in addition to improving information transfer efficiency.
2. By introducing the EMA module, we successfully solve the problem of insufficient integration ability of the ViT model for multi-scale and local features when processing images and significantly enhance the model’s ability to focus on and integrate local details.
3. We conduct a series of comprehensive experiments based on the CottonInsect, IP102, and cotton leaf disease datasets and validate the SpemNet model. The experiments prove that the SpemNet model shows significant effectiveness and superiority in cotton pest and disease identification and has obvious advantages over other strong networks.

## 2. Related Works

Current crop disease recognition methods utilize image processing techniques to extract features; then, the extracted features are used as model inputs for classification using machine learning models. Depending on whether the extraction of features requires manual design of feature engineering, crop disease recognition methods can be categorized into (1) methods based on traditional machine learning and (2) methods based on deep learning.

Traditional machine learning methods need to manually design disease features, such as spectral index, color, texture, etc., then feed them into a classifier for recognition. Rumpf et al. [14] classified healthy and diseased sugar beet leaves based on the spectral index using support vector machine with 97% accuracy. Al et al. [15] proposed a four-phase disease recognition method that includes color preprocessing, cluster segmentation, feature extraction, and shallow neural network classification. These methods perform well in the laboratory, but the accuracy tends to decrease in the natural environment.

In contrast, deep learning-based methods learn features directly from images for classification without the need for manual design. Too et al. [16] compared various deep convolutional neural network (DCNN) models and found that DenseNet121 performed best in terms of accuracy and computational efficiency, reaching 99.75%. Zeng et al. [17] proposed a higher-order residual DCNN, which is more robust to noise and environmental factors. Song et al. [18] proposed a multilevel augmented spatial pyramid network, which achieved 88.4% accuracy on 61 types of pests. Lee et al. [19] compared different DCNNs and achieved 88.4% accuracy on 61 types of pests.

Although these DCNN models are effective, they are limited by local perception and difficult-to-capture high-level semantic features. Therefore, transformer-based visual models (e.g., ViT) have received attention and can better learn long-distance information and semantic information. However, the standard ViT model exhibits poor learning performance from zero on small datasets and needs further optimization. The main optimization methods include (1) image serialization diversification, such as Swin Transformer [20] to introduce hierarchical structures, and (2) attention diversification, such as the introduction of a re-attention mechanism [21]. Although these methods improve performance, they increase the computational requirements. In order to allow the standard ViT model to learn from zero efficiently on small datasets, Lee et al. [22] proposed the rotating chunked sequence (SPT) method, which achieved a performance improvement of 2.96% on Tiny-ImageNet and provided new ideas for crop disease recognition based on transformers.

## 3. Methodology

### 3.1. Overall Network Structure

**Figure 1:**
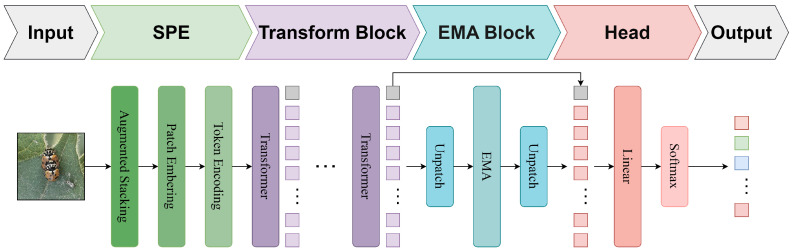
SpemNet receives the original insect images on cotton as input; then, the SPE module extracts local features and adds positional coding. The transformer module processes global and local features, and the EMA module further extracts multi-scale features. Finally, the head layer generates the recognition results of cotton pests and diseases.

The structure of the SpemNet model is shown in Figure 1, including the following six main parts: an input layer, SPE module, transformer module [22], EMA module [13], head layer, and output layer.In the input layer, the insect images on the original cotton are received. In the SPE module, patch features that make it easier to learn local features are obtained by adding biases in different directions, and they are stacked before patch embedding with class tokens and positional encoding. The transformer module uses the standard transformer architecture to process the information obtained from the SPE module. The feature embedding is efficiently extracted and integrated into the global scope to capture the global information and advanced features of local features in the image.

The EMA module introduces the multi-scale feature and parallel sub-network structure to perform multi-scale feature extraction and integrate of the features output from the transformer to obtain feature representations with attention to local details and a stronger integration capability. In the head layer, the feature information output from the EMA module is further integrated, and the feature representation is optimized for the final classification task through the fully connected layer. The final output layer generates the pest and disease recognition results for the input cotton images as specific classification labels indicating the presence or absence of pests on the cotton and the species of pests.

### 3.2. Stacking Patch Embedding

**Figure 2:**
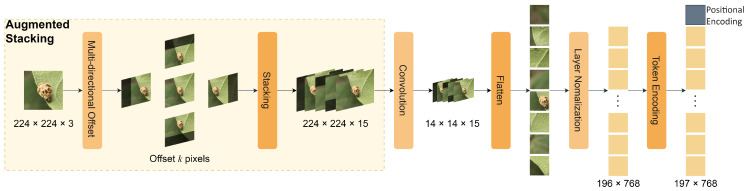
The SPE module first introduces biases in different directions through augmented stacking and enhances the image information through stacking, then uses patch embedding to convert the image features into compact 2D representations through convolution operations and spreading. Finally, the SPE module performs layer normalization on the spread feature maps through token encoding and adds the class token and position encoding.

The structure of the Stacking Patch Embedding (SPE) module is shown in Figure 2. The key role of the Stacking patch embedding module is to solve the problem of loss of inter-block information and difficulty in local feature learning that exist in the traditional Vision Transformer model. Directly dividing the image into patches destroys the spatial relationship between pixels, resulting in the model not being able to fully utilize the local information. The standard ViT model is difficult to mine for a priori knowledge of local spatial relationships in global feature modeling, making it more difficult for the model to learn local features. By introducing the stacking patch embedding module, we are able to effectively improve the model’s ability to learn local features and the efficiency of information transfer so as to better capture the local details in the image and improve the model’s performance.

The stacking patch embedding module consists of the following three key components: augmented stacking, patch embedding, and token encoding. Augmented Stacking enhances the image information by introducing biases in different directions and stacking them, patch embedding transforms image features into compact 2D representations via convolutional operations and spreading, and token encoding performs layer normalization on the spread feature maps and adds class tokens and position encodings.

#### 3.2.1. Augmented Stacking

The purpose of augmented stacking is to enhance the information retention ability of the original image by introducing biases in different directions. Specifically, we construct multiple viewpoints with different orientation biases and supplement the original image information with these differently biased images.

Let the insect image on the original cotton be $X\in R^{H\times W\times C}$, where *H*, *W*, and *C* denote the height, width, and number of channels of the image, respectively. We define *K* bias functions (${\left\{T_{k}\right\}}_{k=1}^{K}$) for different directions, such as up–down bias, left–right bias, oblique bias, etc. By applying these bias functions to the original image (*X*), we can obtain *K* biased images (${\left\{X_{k}\right\}}_{k=1}^{K})$ as follows:

(1) $$X_{k}=T_{k}\left(X\right),\;k=1,2,\ldots ,K$$

Simply put, the specific form of the K-bias function is represented symbolically as $T_{\mathrm{direction}}$. The direction can be divided into different directions, such as up–down, left–right, and diagonal. Each bias function shifts the image by a fixed pixel value (*d*) along the specified direction (*k*). It can be specifically represented as follows:

(2) $$T_{k}\left(X\right)=X+d_{k}$$

where $d_{k}$ is a bias matrix that enhances the information retention capability of an image by introducing biases in specific directions. Specifically, the bias matrix can be implemented by adding displacements in different directions.

(3) $$d_{\mathrm{up}-\mathrm{down}}\left(i,j\right)=\left\{\begin{array}{cc} X(i\pm k,j) & \mathrm{if}\;0\leq i\pm k\leq H\\ 0 & \mathrm{if}\;i\pm k<0\;\mathrm{or}\;i\pm k>H \end{array}\right.$$

(4) $$d_{\mathrm{left}-\mathrm{right}}\left(i,j\right)=\left\{\begin{array}{cc} X(i,j\pm k) & \mathrm{if}\;0\leq j\pm k\leq W\\ 0 & \mathrm{if}\;j\pm k<0\;\mathrm{or}\;j\pm k>W \end{array}\right.$$

(5) $$d_{\mathrm{diagonal}}\left(i,j\right)=\left\{\begin{array}{cc} X(i\pm k,j\pm k) & \mathrm{if}\;0\leq i\pm k\leq H\;\mathrm{and}\;0\leq j\pm k\leq W\\ 0 & \mathrm{otherwise} \end{array}\right.$$

After applying the above operations to the original image (*X*), the biased image is obtained. Stacking the original image (*X*) and the *K* biased images ($X_{k}{\}}_{k=1}^{K}$), we can obtain the enhanced image ($X^{\prime }\in R^{H\times W\times (C\cdot (K+1))}$) as follows:

(6) $$X^{\prime }=\mathrm{Concat}\left(X,X_{1},X_{2},\ldots ,X_{K}\right).$$

#### 3.2.2. Patch Embedding

The main purpose of the patch embedding module is to transform the enhanced image ($X^{\prime }$) into a 2D feature representation suitable for input to the transformer model.

For the enhanced image ($X^{\prime }\in R^{H\times W\times (C\times N)}$, where H=h×P, W=w×P, and M=h×w), $X^{\prime }$ is divided into M×M patches, each with dimensions of P×P×(C×N).

For the *i*th patch ($X_{i}^{\prime }\in R^{P\times P\times (C\times N)}$), a convolutional layer (ϕ) is used for feature extraction to obtain the *D*-dimensional feature vector ($z_{i}\in R^{D}$) as follows:

(7) $$z_{i}=\phi \left(X_{i}^{\prime }\right)$$

Then, all $M^{2}$ eigenvectors (${\left\{z_{i}\right\}}_{i=1}^{M^{2}}$) are into an eigenmatrix ($Z\in R^{M^{2}\times D}$) as follows:

(8) $$Z={\left[z_{1},z_{2},...,z_{M^{2}}\right]}^{⊤}$$

Subsequently, layer normalization is applied to the feature matrix (Z) as follows:

(9) $$\hat{Z}=\mathrm{LayerNorm}\left(Z\right)$$

In this way, the patch embedding module transforms the original augmented image ($X^{\prime }$) into a compact 2D feature representation ($\hat{Z}$), providing suitable inputs for the subsequent token encoding and transformer modules.

#### 3.2.3. Token Encoding

The the main purpose of the token encoding module is to add category encoding and location encoding information to the feature matrix $\hat{z}$ obtained by the patch embedding module to make the output more suitable for input into the transformer model.

Then, a category encoding ($c_{i}\in R^{C}$, where *C* is the number of categories) is added to the feature vector (${\hat{z}}_{i}\in R^{D}$) of each patch. Splicing the category encoding with the feature vector (${\hat{z}}_{i}$) yields the augmented feature vector ($x_{i}\in R^{D+C}$):

(10) $$x_{i}=\left[{\hat{z}}_{i},c_{i}\right]$$

Subsequently, a position encoding ($p_{i}\in R^{D+C}$) is added to the augmented feature vector ($x_{i}\in R^{D+C}$) for each patch, where *P* is the position encoding dimension.

The position encoding is summed with the augmented feature vector ($x_{i}$) to obtain the final token representation ($t_{i}\in R^{D+C+P}$) as follows:

(11) $$t_{i}=x_{i}+p_{i}$$

### 3.3. Transformer Block

The transformer block [22] is the main part of the SpemNet model and is responsible for global feature extraction and synthesis of the feature embeddings obtained from the SPE module. This module adopts the standard transformer architecture and contains multiple transformer encoder layers, which are designed to efficiently capture global information and complex high-level features from the input features through the self-attention mechanism and feed-forward neural networks. Compared with traditional convolutional neural network, the transformer architecture has stronger modeling ability and can better learn the long-range dependencies in the image. In SpemNet, we use 12 stacked transformer encoder layers, which enables the model to learn richer and more abstract feature representations in a deeper network structure. Through the stacking of multiple transformer layers, the model can gradually extract multi-scale features from local to global, providing more effective feature inputs for the subsequent EMA module and classification tasks.

Each transformer contains self-attention mechanisms, feed-forward neural networks, and layer normalization, and these components work together to progressively map the encoded features to the final output.

Self-attention mechanism: By calculating the attention weights between features, the model is able to understand the interrelationships between points and, thus, globally optimize the feature representation. The computation of the self-attention mechanism can be expressed as follows:

(12) $$\mathrm{SelfAttention}\left(Q_{\mathrm{self}},K_{\mathrm{self}},V_{\mathrm{self}}\right)=\mathrm{softmax}\left(\frac{Q_{\mathrm{self}}K_{\mathrm{self}}^{T}}{\sqrt{d_{k}}}\right)V_{\mathrm{self}}$$

In the self-attention mechanism, the query matrix (*Q*), key matrix (*K*), and value matrix (*V*) all come from the same sequence of feature representations, where $Q_{\mathrm{self}}=XW_{Q}$; $K_{\mathrm{self}}=XW_{K}$; $V_{\mathrm{self}}=XW_{V}$; *X* is the input feature representation; and $W_{Q}$, $W_{K}$, and $W_{V}$ are learnable weight matrices.

The cross-attention mechanism is similar to self-attention, with the difference being that it deals with the relationship between two different sequences. In this mechanism, elements of one sequence (e.g., a query) are used to pay attention to elements of another sequence (e.g., keys and values), enabling the model to capture correlations between different data sources. The formula is expressed as follows:

(13) $$\mathrm{CrossAttention}\left(Q_{\mathrm{target}},K_{\mathrm{source}},V_{\mathrm{source}}\right)=\mathrm{softmax}\left(\frac{Q_{\mathrm{target}}K_{\mathrm{source}}^{T}}{\sqrt{d_{k}}}\right)V_{\mathrm{source}}$$

In the cross-attention mechanism, the query matrix (*Q*) comes from one sequence (usually the target sequence) of feature representations, while the key matrix (*K*) and value matrix (*V*) come from another sequence (usually the source sequence) of feature representations, where $Q_{\mathrm{target}}=YW_{Q}$; $K_{\mathrm{source}}=ZW_{K}$; $V_{\mathrm{source}}=ZW_{V}$; *Y* is the input feature representation of the target sequence; *Z* is the input feature representation of the source sequence; and $W_{Q}$, $W_{K}$, and $W_{V}$ are learnable weight matrices.

A feed-forward neural network (FFN) is a component within a transformer that typically consists of two layers of linear transformations and a nonlinear activation function. The FFN processes features independently for each location, increasing the model’s ability to handle different feature representations. The formula is expressed as follows:

(14) $$\mathrm{FFN}\left(x\right)=\max\left(0,xW_{1}+b_{1}\right)W_{2}+b_{2}$$

where $W_{1},W_{2}$, $b_{1}$, and $b_{2}$ are network parameters and ReLU(max(0,x)) is the nonlinear activation function.

Layer normalization is the process of normalizing each sample over all features, aiming to reduce the internal covariate bias during training and stabilize the learning of the deep network. In the transformer, it is usually applied after self-attention and FFN. The formula is expressed as follows:

(15) $$\mathrm{LayerNorm}\left(x\right)=\gamma \left(\frac{x-\mu }{\sqrt{\sigma^{2}+\epsilon }}\right)+\beta$$

where *x* is the input; μ and $\sigma^{2}$ are the mean and variance of the input, respectively; γ and β are learnable parameters; and ϵ is a small constant added for numerical stability.

Residual concatenation allows the inputs of a model to be added directly to its outputs, helping to mitigate the problem of vanishing gradients during deep network training. In the transformer, the output of each sublayer (self-attention layer or FFN) is added to the input of that sublayer, which is then layer normalized.

### 3.4. Efficient Multi-Scale Attention Block

**Figure 3:**
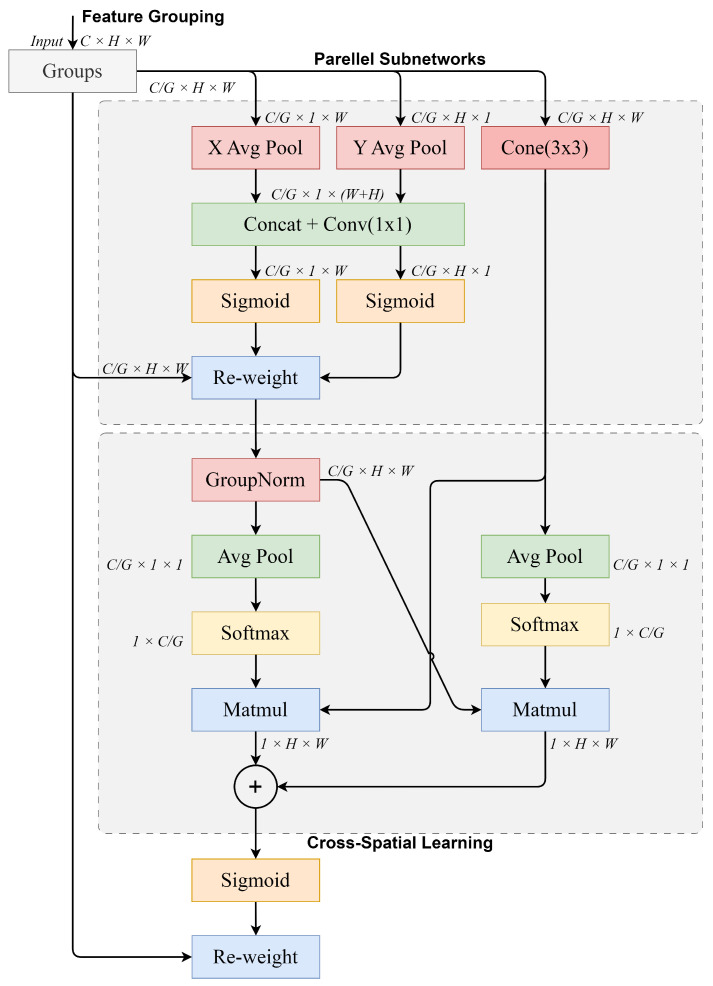
First, the EMA processes the input features in groups. Subsequently, new features are generated through parallel sub-networks by processing each group of features through different paths. In the cross-space learning stage, the features are integrated using weight assignment to effectively integrate cross-space information.

The Efficient Multi-Scale Attention (EMA) block [13] is shown in Figure 3, which is another key component in the SpemNet model designed to solve the problem of the Vision Transformer model’s insufficient ability to integrate multi-scale and local features when processing images. In the traditional ViT model, due to the adoption of a single self-attention mechanism, it is difficult to effectively capture and integrate features at different scales in an image, resulting in the model’s insufficient ability to pay attention to and integrate local detailed features. To overcome this problem, we introduce the EMA module in SpemNet. This module adopts the multi-scale feature and parallel sub-network structure, which significantly enhances the model’s ability to focus on and integrate local details by simultaneously extracting and fusing features of different scales.

Imagine you are looking at an image with many details, such as a complex photo of a cotton field. You not only need to pay attention to the overall layout (e.g., the panorama of the cotton field) but also focus on smaller details (e.g., pests on a single cotton leaf). The EMA module is like a magnifying glass that can handle both the overall and local details of the image, allowing the model to better identify important features in the image.

Specifically, the EMA module contains multiple parallel attention sub-networks, each focusing on different scales of feature extraction. In the feature fusion stage, the EMA module integrates these different scales of features through weighted summation to generate the final feature representation. This multi-scale feature fusion approach enables the model to better focus on the key regions and detailed features in the image, providing richer and more effective feature inputs for the subsequent classification task.

The EMA module consists of the following three main components: feature grouping, a parallel sub-network, and cross-space learning. In the feature grouping stage, the feature representations obtained from the transformer block are divided into multiple groups, and the features in each group undergo different processing paths. In the parallel sub-network stage, each group of features undergoes average pooling in the X and Y directions; 3 × 3 convolution operations; and, finally, splicing and convolution to generate a new feature representation. The cross-space learning stage performs GroupNorm processing on the features, generates a weight map through average pooling and softmax operations, and achieves cross-space weight assignment through matrix multiplication.

In the efficient multi-scale attention (EMA) module, the feature grouping stage first divides the feature representations ($F\in R^{C\times H\times W}$) from the transformer block into *G* groups, with the size of each group being $F_{g}\in R^{(\frac{C}{G})\times H\times W}$. Next, each group of features goes through three different processing paths.

The first two paths perform average pooling operations on each group of features in the horizontal and vertical directions, respectively, to obtain $F_{xavg}\in R^{(\frac{C}{G})\times 1\times W}$ and $F_{yavg}\in R^{(\frac{C}{G})\times H\times 1}$. These two feature maps are processed by a 1×1 convolutional layer and Sigmoid activation function, respectively, to obtain weighted feature representations of $W_{x}\in R^{(\frac{C}{G})\times 1\times W}$ and $W_{y}\in R^{(\frac{C}{G})\times H\times 1}$.

The third path performs a 3×3 convolution operation on each set of features to obtain another feature map ($F_{conv}\in R^{(\frac{C}{G})\times H\times W}$).

The output feature maps of these three paths are spliced and passed through a 1×1 convolutional layer to obtain a new feature representation ($F_{new}\in R^{(\frac{C}{G})\times H\times W}$).

In the cross-space learning phase, a group normalization operation is first performed on the new feature representation ($F_{new}$) to obtain the normalized features ($F_{norm}\in R^{(\frac{C}{G})\times H\times W}$). Next, an average pooling operation in the X and Y directions is performed on $F_{norm}$ to obtain $F_{xpool}\in R^{(\frac{C}{G})\times 1\times W}$ and $F_{ypool}\in R^{(\frac{C}{G})\times H\times 1}$, respectively. These two feature maps are subjected to a softmax operation to obtain weight maps of $W_{x}\in R^{(\frac{C}{G})\times 1\times W}$ and $W_{y}\in R^{(\frac{C}{G})\times H\times 1}$, respectively.

Finally, $W_{x}$ and $W_{y}$ are multiplied by $F_{norm}$ to obtain the weighted feature representation ($F_{weighted}\in R^{(\frac{C}{G})\times H\times W}$). A Re-weighting operation is performed by a Sigmoid activation function to obtain the final multi-scale feature representation ($F_{final}\in R^{C\times H\times W}$), which is passed to the head layer for further processing and classification tasks.

### 3.5. Classification Head

The classification head module receives multi-scale feature representations from the EMA block and processes these features for dimensionality reduction and integration through a fully connected layer to achieve better feature representation and differentiation capabilities. The role of the full connectivity layer is to establish the global connection between different features, which helps to realize the nonlinear combination of features, thereby highlighting the important features.

Subsequently, the features passing through the fully connected layer are mapped to a new feature space, and the nonlinear representation of the features is enhanced by activation functions to further optimize the feature representation.

Finally, after softmax processing in the classification decision layer, the optimized features are transformed into probability distributions for each category, thereby achieving recognition of insect images or cotton leaf disease images on input cotton. By selecting the category with the highest probability as the final prediction, the classification head module generates the corresponding classification labels, indicating the presence of pests on cotton and the species of the pests.

### 3.6. Loss Function

In the SpemNet cotton pest and disease recognition network, the loss function uses multi-class cross-entropy loss to measure the difference between the model output and the actual labels, guiding the optimization of the network parameters to achieve accurate disease recognition. Since the goal of SpemNet is cotton pest and disease recognition, targeting both cotton pests and diseases of cotton leaves, the multi-class cross-entropy loss for each classification head can be expressed as follows:

(16) $${\mathrm{Loss}}_{\mathrm{pest}}=-\frac{1}{N_{\mathrm{pest}}}\sum_{i=1}^{N}\sum_{c=1}^{C_{\mathrm{pest}}}y_{i,c}\log\left(p_{i,c}\right)$$

(17) $${\mathrm{Loss}}_{\mathrm{disease}}=-\frac{1}{N_{\mathrm{disease}}}\sum_{i=1}^{N}\sum_{c=1}^{C_{\mathrm{disease}}}y_{i,c}\log\left(p_{i,c}\right)$$

(18) $$\mathrm{Total}\;\mathrm{Loss}=\alpha \cdot {\mathrm{Loss}}_{\mathrm{pest}}+\beta \cdot {\mathrm{Loss}}_{\mathrm{disease}}$$

where *N* is the number of samples, *C* is the number of classes, $y_{i,c}$ is the true label of the *i*-th sample for class *c* (one-hot encoded), $p_{i,c}$ is the predicted probability of the *i*-th sample for class *c*, and α and β can be assigned different weights according to the needs of the task. If the insect identification task is more complex than the plant disease identification task, you might want to assign a larger weight to the insect identification task to ensure that the model pays more attention to this more complex task during training. This can be achieved by adjusting the weight parameters in the loss function.

## 4. Experiments

### 4.1. Experimental Datasets

**Figure 4:**
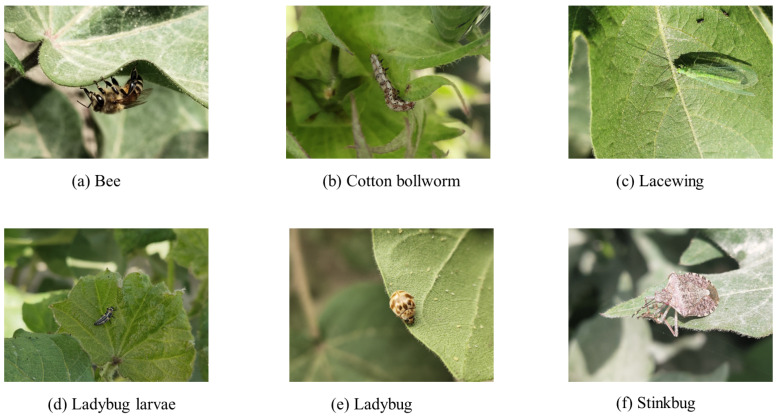
Examples of common cotton field insects.

In order to verify the superiority of the SpemNet model in the field of pest recognition, image data provided in the publicly available CottonInsect dataset [22,23] were used in this experiment. This dataset is specially designed for the problem of classifying and recognizing insects in cotton fields and contains images of a variety of common pests, such as cotton bollworm, polyisopteranladybug beetle, and alfalfa blind stink bug. It contains a total of 3225 high-quality images, each containing one or more insects of the same species type, showing the characteristics of these pests in different states of growth and activity. Figure 4 shows some examples of common cotton field insects in the CottonInsect dataset.

**Figure 5:**
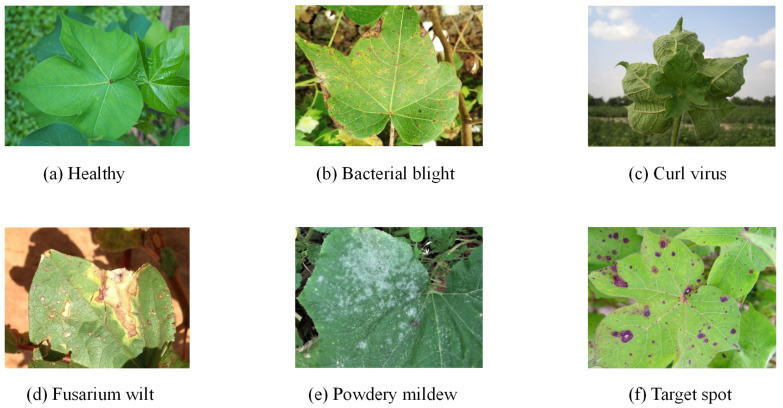
Images of cotton leaf disease samples.

Due to the diversity of pest species in agricultural fields, it is not sufficient to discuss specific pests in isolation. Farmlands can be infested not only by specific pests but also by many common pests; therefore, a wide range of possible pests needs to be identified and classified. IP102 [24] is a more extensive insect image dataset than the CottonInsect dataset, containing 75,820 high-quality images covering 102 different insect classes, including common pests and beneficial insects in agriculture and forestry. Experiments were conducted to extract 13 classes from the dataset based on common pests in cotton fields, totaling 5314 images. Figure 5 shows some examples of common leaf diseases of cotton in reality.

The above dataset is primarily used for insect identification in cotton fields. However, to achieve integrated identification of cotton pests and diseases, in this study, we further identified cotton diseases. To this end, we collected a large amount of cotton disease image data in actual cotton fields and combined them with web-crawled data to construct the dataset. This dataset covers six common categories of cotton leaf diseases, totaling 5348 high-quality images and providing rich sample data for model validation.

**Figure 6:**
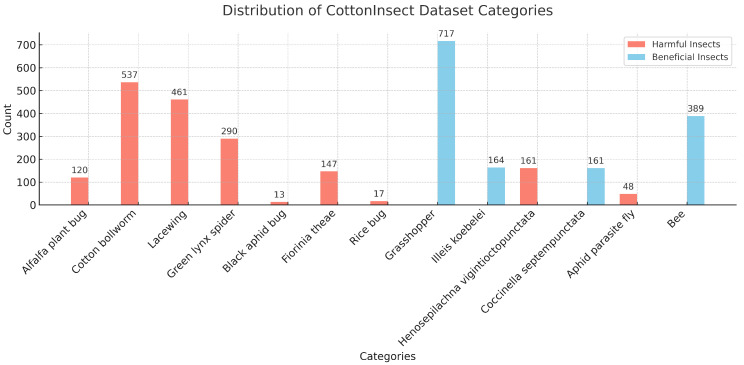
Distribution of CottonInsect dataset categories.

**Figure 7:**
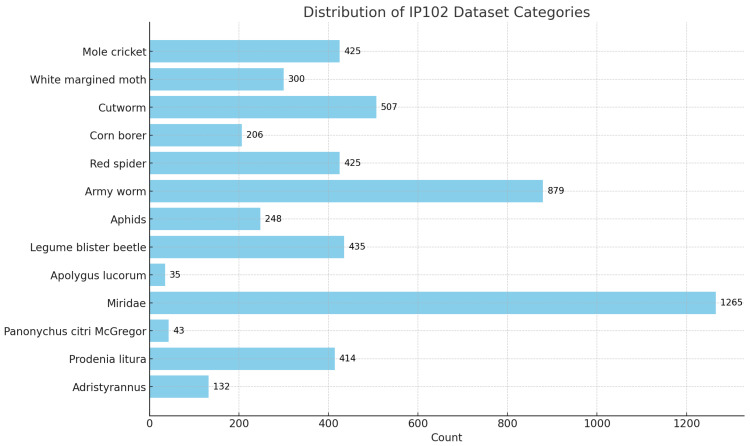
Distribution of IP102 dataset categories.

**Figure 8:**
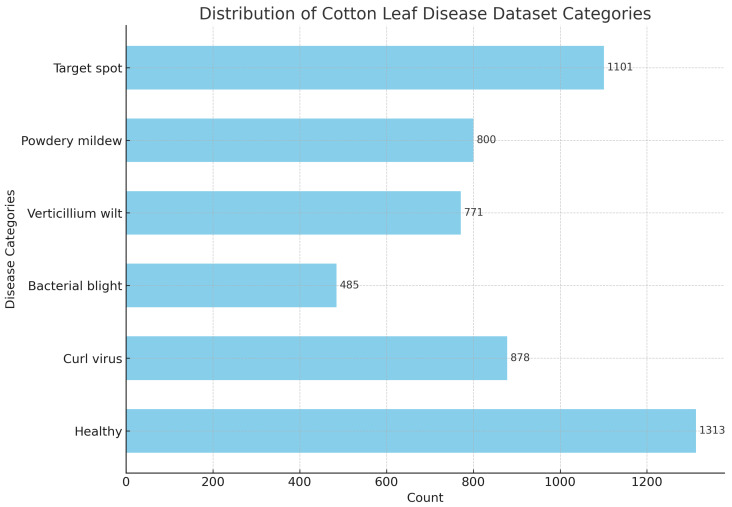
Distribution of cotton leaf disease dataset categories.

The distribution of dataset categories is shown in Figure 6, Figure 7 and Figure 8, with the training set, validation set, and test set divided in a ratio of 7:1:2.

### 4.2. Evaluation Indicators

In order to comprehensively reflect the performance improvement of the SpemNet method in the cotton pest and disease recognition task, several evaluation metrics are introduced in this study. Given that the identification method used in this study is a classification task, three commonly used regression metrics were used in this study, namely precision, recall, and F1 score.

(19) $$\mathrm{Precision}=\frac{\mathrm{TP}}{\mathrm{TP}+\mathrm{FP}}$$

(20) $$\mathrm{Recall}=\frac{\mathrm{TP}}{\mathrm{TP}+\mathrm{FN}}$$

(21) $$F_{1}=2\times \frac{\mathrm{Precision}\times \mathrm{Recall}}{\mathrm{Precision}+\mathrm{Recall},}$$

where TP (True Positive) represents the number of samples that are actually in the positive category and predicted by the model to be in the positive category, TN (True Negative) represents the number of samples that are actually in the negative category and predicted by the model to be in the negative category, FP (False Positive) represents the number of samples that are actually in the negative category but predicted by the model to be in the positive category, and FN (False Negative) is the number of samples that are actually negative but predicted to be positive by the model.

### 4.3. Experimental Settings

The experimental setup of this paper includes the experimental environment and parameter settings, as shown in Table 1 and Table 2:

The model was trained using the AdamW optimizer with a training batch size of 8 and a test batch size of 32. The initial learning rate was set to 0.00001, and the weights decayed to 0.01. A learning rate tuning strategy with warm-up was used, whereby the learning rate was gradually increased in the first few epochs to reach 0.0001, then progressively decayed at a rate of 0.8, (but not lower than 0.00001).

The input image is resized to 224 × 224 pixels; then, the resized image is divided into multiple 16 × 16 pixel patches. Each patch corresponds to a small block of the image, and these small blocks are fed into the model for further processing. A patch_bias of 6 indicates that in the SPE module, each patch is shifted by six pixels in different directions. An in_c value of 3 indicates that the model expects the input image to be an RGB image with three channels.

The model has an embedding dimension of 768, meaning each patch is converted into a 768-dimensional vector after passing through the embedding layer. It uses a transformer encoder with 12 layers to progressively extract and integrate image features. The multi-head self-attention mechanism has eight heads.

### 4.4. Comparison Experiment

In this section, we compare the performance of the SpemNet model with several popular benchmark methods, including SVM [25], VGG [26], ResNet [27], EfficientNet_v2 [28,29], ConvNeXt [30], MobileViT_v2 [31,32], ShuffleNet_v2 [33,34], Swin Transformer [35], and Vision Transformer [12]. We evaluated the pest recognition performance of each method on three datasets and quantitatively assessed it by calculating precision, recall, and F1 score. Table 3 and Table 4 list the performance of each method in the pest recognition task.

On the CottonInsect dataset, SpemNet significantly outperforms other models. It achieves a precision of 99.03%, a recall of 98.67%, and an F1 score of 0.9885. In comparison, the Swin_transformer model, while slightly higher in precision, at 99.45%, has a lower recall of 97.59% and an F1 score of 0.9851. Other models, such as Vision_transformer and Convnext, also exhibit high precision and recall but do not surpass the overall performance of our model.

On the IP102 dataset, SpemNet, again, demonstrates superior performance, with a precision of 95.35%, a recall of 95.68%, and an F1 score of 0.9551. In contrast, the Swin_transformer model shows a precision of 93.29%, a recall of 94.01%, and an F1 score of 0.9365. The Vision_transformer model achieves a precision of 93.43% and a recall of 94.70%, with an F1 value of 0.9406. Other models, like ShuffleNet_v2, while performing well, fall short in precision, recall, and F1 score compared to SpemNet, indicating that SpemNet achieves the best overall performance on this dataset.

**Figure 9:**
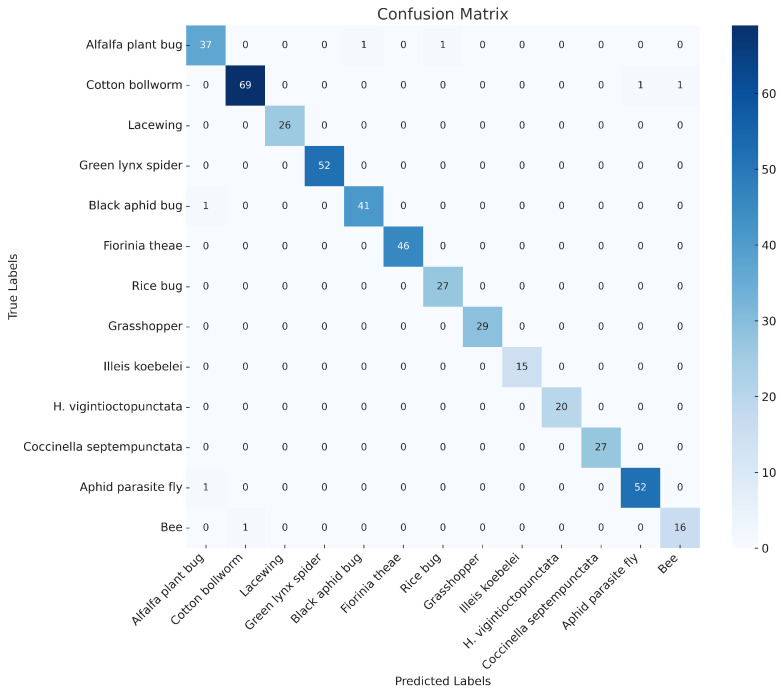
SpemNet confusion matrix results on the CottonInsect dataset.

**Figure 10:**
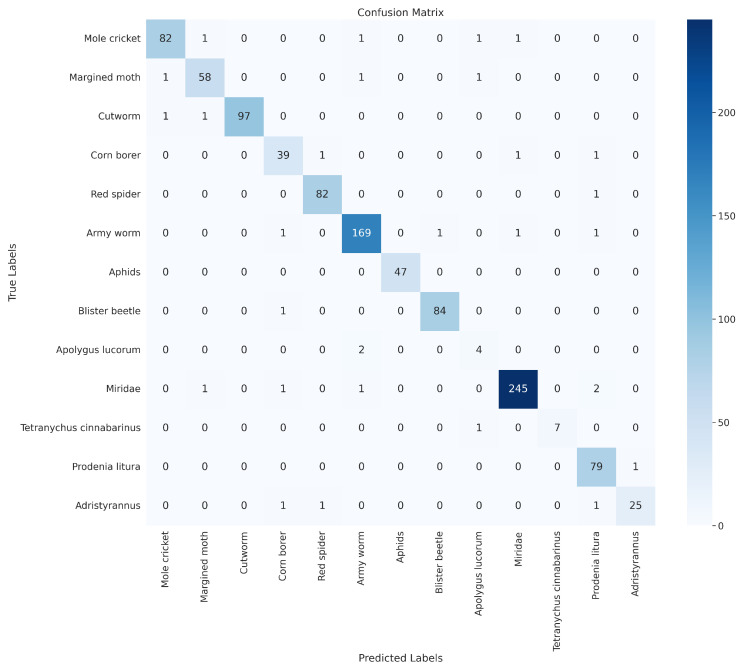
SpemNet confusion matrix results on the IP102 dataset.

The confusion matrix in Figure 9 and Figure 10 shows the classification results of the SpemNet model on the CottonInsect dataset and the IP102 dataset, and the results of the confusion matrix show that SpemNet performs satisfactorily and achieves excellent results on all the classified data.

Table 4 demonstrates the performance of the SpemNet model on cotton leaf disease data. The SpemNet model excels in three main metrics. Its precision is 94.87%, which is significantly higher than that of Vision_transformer (92.08%) and Swin_transformer (91.96%). In terms of recall, SpemNet’s 94.70% is higher than Vision_transformer’s 91.73% and Swin_transformer’s 91.41%. SpemNet has high F1 score of 0.9479, which is well ahead of that of Vision_transformer (0.9190) and Swin_transformer (0.9168).

**Figure 11:**
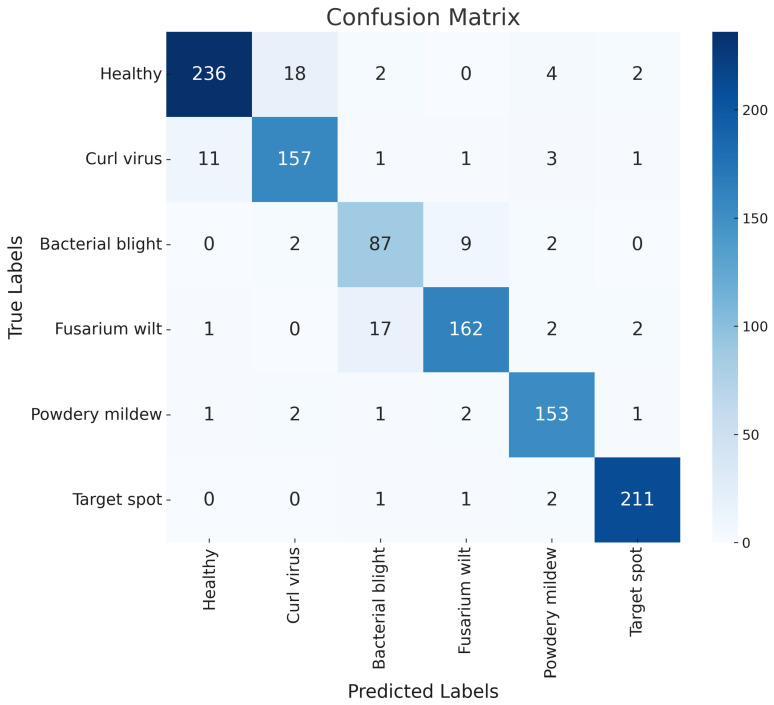
Confusion matrix results of SpemNet on cotton leaf disease data.

The confusion matrix in Figure 11 demonstrates the classification results of the SpemNet model in recognizing cotton leaf diseases. The precision of cotton leaf disease classification is decreased compared to pest classification, possibly due to the distinctive features of pests compared to the less distinctive features of cotton leaf diseases. In pest picture classification, morphological, color, and structural features of pests are usually very distinctive and can be identified and classified more easily by the model. However, the symptoms of cotton leaf diseases are more subtle in the early stages and in cases of mild infections; spots, discoloration, and shape changes on the leaves are often not obvious; and the symptoms of different diseases can be easily confused owing to their similarities. For example, leaf curl virus and healthy leaves may show similar leaf curling and color changes in some cases, early symptoms of bacterial spots and other diseases such as watery spots may be difficult to distinguish, and spot characteristics of powdery mildew and target spot may not be obvious under different light conditions or in the early stages of infection, further increasing the difficulty of classification.

Comprehensive evaluation shows a high level of overall performance of SpemNet.SpemNet shows great potential and superiority in coping with the task of cotton leaf pest and disease identification, making it worthy of further in-depth research and application.

### 4.5. Ablation Experiments

#### 4.5.1. Overall Ablation Experiment

**Figure 12:**
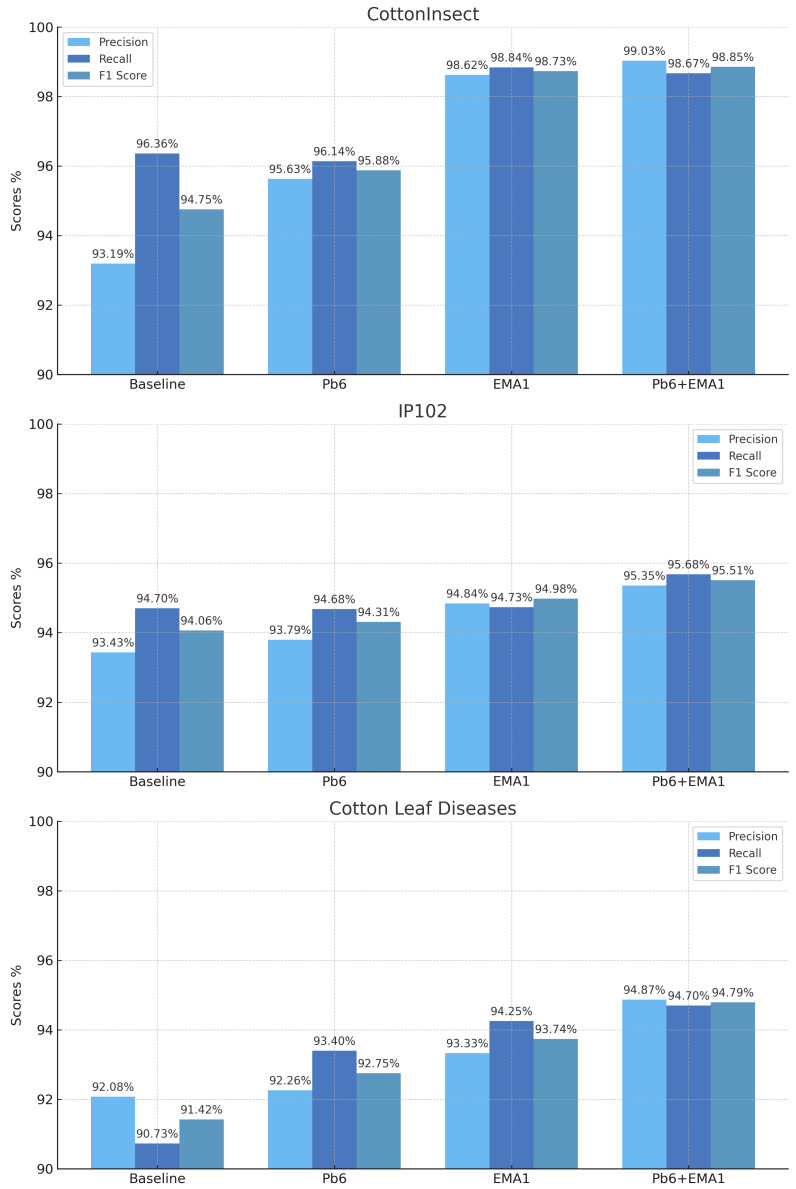
Results of ablation experiment.

In order to validate the effectiveness of individual modules in the SpemNet model, we conducted detailed ablation experiments. By gradually removing or replacing specific modules in the model, we evaluated the impact of each module on model performance. The results of the experiments are shown in Figure 12.

We conducted ablation experiments on the CottonInsect dataset, testing each of the following model configurations:

1. Baseline: the original ViT model;
2. Pb6: introducing the SPE module in the ViT model, with patch bias set to 6 px;
3. EMA1: introducing the EMA module in the ViT model using one EMA attention layer after the transformer block;
4. Pb6+EMA1: introducing both the SPE module (with patch bias set to 6 px) and the EMA module (one EMA attention layer) in the ViT model.

The experimental results presented in Figure 12 show that the baseline methodology provides a solid foundation across all datasets, while the introduction of the Pb6 module significantly improves accuracy and F1 scores. This suggests that the Pb6 module effectively enhances the capabilities of the model in specific aspects. In addition, the implementation of the EMA1 module resulted in significant improvements in all metrics, with peak F1 scores of 0.9873, 0.9498, and 0.9374 on the CottonInsect, IP102, and cotton leaf disease datasets, respectively, highlighting the module’s important role in enhancing the integration of multiscale and localized features. The combination of Pb6 and EMA1 modules, represented by the Pb6+EMA1 method, achieved the best performance on key metrics such as precision, recall, and F1 score across all datasets, with scores as high as 0.9885, 0.9551, and 0.9479, respectively. These findings confirm the strong enhancement capabilities of the Pb6 and EMA1 modules, which make them useful for improving performance in different agricultural environments in the context of identification tasks (e.g., cotton pest and disease identification).

#### 4.5.2. Patch Bias Ablation Experiment

**Figure 13:**
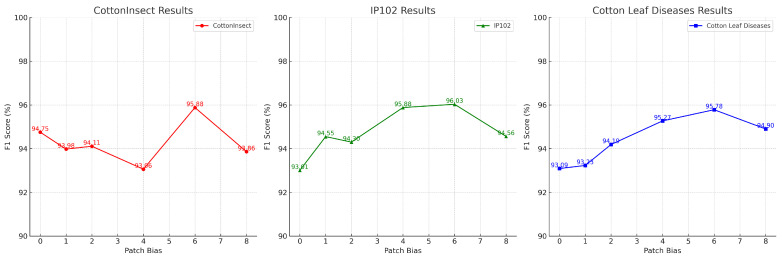
Results of patch bias ablation experiment.

In order to evaluate the effect of different orientations of bias in the SPE module on model performance, we conducted an ablation experiment to specifically analyze the effect of different patch bias offsets on model performance on three datasets. In this ablation experiment, we set up different experimental groups with patch bias offsets of 0, 1, 2, 4, 6, and 8 and performed model training and testing on the three datasets. The experimental results are shown in Figure 13.

The experimental results show that patch bias has a significant effect on model performance. With a patch bias of 0, the model’s precision, recall, and F1 score perform poorly, pointing out that the model has challenges in learning local features. With an increase in patch bias, the model performance gradually improves, with peak performance reached at a patch bias of 6, indicating that a moderate bias helps to improve the model’s learning of local features, as well as its information transfer efficiency. However, too of large a bias (e.g., patch bias of 8) leads to performance degradation and may introduce noise, affecting the feature learning ability of the model. Based on the experimental results, we suggest choosing a moderate patch bias offset to balance the relationship between local feature learning and noise introduction in practical applications.

#### 4.5.3. EMA Layer Ablation Experiment

**Figure 14:**
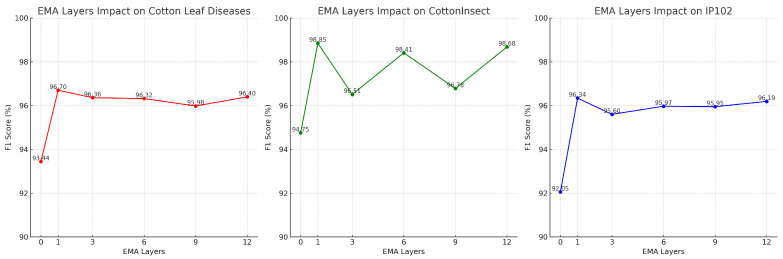
Results of the EMA layer ablation experiment.

In order to evaluate the effect of the number of EMA module layers on the performance of the SpemNet model, we conducted an ablation experiment in the cotton pest identification task. In this experiment, we set up 0, 1, 3, 6, 9, and 12 layers of EMA modules and conducted comprehensive tests on three datasets. The experimental results are shown in Figure 14.

The figure clearly demonstrates that as the number of EMA layers increases, the F1 scores for the CottonInsect, IP102, and cotton leaf disease datasets show different trends. For the CottonInsect dataset, increasing the number of EMA layers significantly improves the F1 score compared to the initial setting of 0 layers, especially at 1 and 6 layers, where the scores approach their highest values of 98.85 and 98.41 respectively. Similarly, the IP102 dataset also shows an improvement in F1 score with an increase in the number of EMA layers, peaking at one layer, with a score of 96.34. However, for the cotton leaf disease dataset, while increasing the number of EMA layers also enhances the F1 score, the overall gain is relatively steady, with the highest point also occurring with one layer, with a score of 96.70. Overall, increasing the number of EMA layers positively affects model performance, as appropriately increasing the number of EMA layers can effectively enhance the model’s recognition accuracy. However, too many EMA layers lead to resource consumption without substantial improvements, possibly due to the insufficient complexity of the datasets. If the datasets were more complex, perhaps better performance could be observed.

## 5. Conclusions

In this study, we propose a cotton pest and disease recognition method, SpemNet, based on efficient multi-scale attention and stacking patch embedding. By introducing the SPE module and the EMA module, we successfully solve the problems of difficult local feature learning and insufficient multi-scale feature integration in the traditional Vision Transformer model and significantly improve the performance and efficiency of the model. In experiments, we comprehensively validated the SpemNet model using the CottonInsect and IP102 datasets, as well as our own collected dataset. The experimental results demonstrate that SpemNet performs excellently in the task of cotton pest identification, showing significant effectiveness and superiority. Compared to a variety of strong baseline methods, SpemNet exhibits advantages in key metrics such as precision, recall, and F1 score, demonstrating its great potential in practical applications. However, SpemNet still has some shortcomings, which are summarized as follows:

(1) In actual field environments, the identification of cotton pests and diseases faces challenges far more complex than those in laboratory settings. For example, field lighting conditions, background interference, and weather factors such as rain and fog can significantly affect the quality of images and the accuracy of identification. Additionally, the manifestations of pests and diseases can vary due to geographic location, cultivation methods, and cotton varieties, necessitating a model with higher adaptability and robustness.
(2) In practical applications such as multi-output classification tasks, the SpemNet model often faces challenges more complex than in single-output classification tasks. For instance, an image may contain multiple objects simultaneously, and the limitations of the SpemNet model may lead it to favor the most prominent feature in an image with multiple objects. This bias can restrict the model’s ability to recognize, causing it to fail to comprehensively reflect all target categories in the image at once.

In response to the aforementioned deficiencies, future research will consider integrating multi-source data to enhance the model’s generalization capabilities across different geographic locations, cultivation methods, and cotton varieties (https://github.com/Xnightwish/Cotton-Leaf-Pest-Disease-Recognition (accessed on 6 August 2024)). In addition, there are future plans to improve the existing SpemNet model for multi-label recognition, e.g., by combining migration learning and multi-task learning or by utilizing target detection frameworks such as YOLO to deal with the task of handling multiple target categories in an image so as to ensure its effectiveness and stability in real-world applications.

## Figures and Tables

**Table 1:** Configuration of the experimental environment.

Experimental Environment	Environmental Configuration
System	Linux
GPU	RTX 4090
CPU	16 vCPU Intel(R)
	Xeon(R) Platinum 8352V
Pytorch	1.11.0
Python	3.8
Cuda	11.3

**Table 2:** Model parameters and settings.

Parameter	Parameter Setting
Epoch	300
Learning_Rate	0.00001
Optimizer	AdamW
Dropout	0.5
weight_decay	0.01
Train_batch_size	8
Test_batch_size	32
img_size	224
patch_size	16
patch_bias	6
in_c	3
embed_dim	768
Transformer_Block_Layers	12
EMA_Layers	1
num_multi_heads	8

**Table 3:** Experimental results of pest identification with different models.

Model	CottonInsect			IP102		
	Precision	Recall	F1	Precision	Recall	F1
SVM	53.37	69.10	0.6022	50.02	70.38	0.5854
VGG	79.61	76.80	0.7818	80.34	76.47	0.7834
ResNet	85.66	87.47	0.8656	85.56	83.12	0.8427
Efficientnet_v2	89.36	96.66	0.9287	87.72	92.13	0.8982
Convnext	94.61	88.27	0.9133	90.31	85.29	0.8770
Mobilevit_v2	90.22	97.63	0.9378	88.47	80.24	0.8415
ShuffleNet_v2	97.43	94.58	0.9598	90.12	90.47	0.9029
Swin_transformer	**99.45**	97.59	0.9851	93.29	94.01	0.9365
Vision_transformer	93.19	96.36	0.9475	93.43	94.70	0.9406
Ours	99.03	**98.67**	**0.9885**	**95.35**	**95.68**	**0.9551**

**Table 4:** Experimental results of different models for the identification of six classes of leaf pests and diseases on cotton leaves.

Model	Precision	Recall	F1
SVM	64.61	75.52	0.6953
VGG	82.50	86.39	0.8433
ResNet	87.55	90.63	0.8906
Efficientnet_v2	82.25	83.55	0.8276
Convnext	87.50	85.16	0.8617
Mobilevit_v2	88.11	90.90	0.9006
ShuffleNet_v2	90.31	87.74	0.8894
Swin_transformer	91.96	91.41	0.9168
Vision_transformer	92.08	91.73	0.9190
Ours	**94.87**	**94.70**	**0.9479**

## Data Availability

The data that support the findings of this study are available from the authors upon reasonable request.
